# The translation of cancer genomics: time for a revolution in clinical cancer care

**DOI:** 10.1186/gm539

**Published:** 2014-03-26

**Authors:** Elaine R Mardis

**Affiliations:** 1 The Genome Institute, Washington University School of Medicine, 4444 Forest Park Blvd, St Louis, MO 63108, USA

## Abstract

The introduction of next-generation sequencing technologies has dramatically impacted the life sciences, perhaps most profoundly in the area of cancer genomics. Clinical applications of next-generation sequencing and associated methods are emerging from ongoing large-scale discovery projects that have catalogued hundreds of genes as having a role in cancer susceptibility, onset and progression. For example, discovery cancer genomics has confirmed that many of the same genes are altered by mutation, copy number gain or loss, or structural variation across multiple tumor types, resulting in a gain or loss of function that likely contributes to cancer development in these tissues. Beyond these frequently mutated genes, we now know there is a ‘long tail’ of less frequently mutated, but probably important, genes that play roles in cancer onset or progression. Here, I discuss some of the remaining barriers to clinical translation, and look forward to new applications of these technologies in cancer care.

## Introduction

In 2004, following the completion of the Human Genome Project [1], a correlation was described between a specific set of mutations and patients whose lung cancers had dramatically responded to a new class of therapeutics called tyrosine kinase inhibitors (TKIs) [2]. Our group and two others [2-4] found that about 80% of patients with a response to a TKI carried somatic mutations in the gene encoding the epidermal growth factor receptor (EGFR) resulting in changes to the amino acid sequence. Those efforts involved low-throughput technology that combined PCR amplification of the *EGFR* exons with fluorescent capillary electrophoresis-based sequencing, and yet were revealing in that they linked a specific gene and its mutations to a specific therapy and to a patient’s response. Exciting as these discoveries were just 8 years ago, today the first-line therapy and standard of care for non-small-cell lung cancer patients with mutated *EGFR* is to prescribe a TKI. While these mutations occur in only about 10% of lung adenocarcinoma patients, subsequently other genes with corresponding targeted therapies have been identified in lung cancer. Furthermore, a large number of driver alterations have been identified in lung adenocarcinoma, a significant proportion of which can be treated with specific drug-gene combinations, and there are many more drivers to be identified [5].

In the ensuing 8 years, DNA sequencing technology has taken a dramatic and welcome turn away from those expensive and slow methods of 2004, to so-called ‘next-generation’ or ‘massively parallel’ technologies that can deliver DNA sequencing data for a complete human genome overnight. The result of this technology acceleration, and the accompanying types of sophisticated analyses that can be applied to cancer genome data, have transformed our understanding of the numbers and types of somatic alterations that drive cancer development [6]. Importantly, not only can rapid DNA sequencing now quickly identify mutated genes, but these can be matched to increasing numbers of protein-targeted therapies, creating a virtuous circle that is beginning to revolutionize clinical cancer care. However, there are issues that complicate the application of our new knowledge to the care of cancer patients. These issues revolve around the difficulties of adding new methods to already established standards of care in medicine, the need to train physicians across the cancer care discipline regarding how best to apply these new data to clinical practice, and how to make the enhanced information from genomics ‘fit’ into a drug approval process that has, until now, only dealt with drugs targeting single disease sites.

## Debatable issues for translation

A multitude of questions remain unanswered in the clinical translation of next-generation sequencing (NGS) for therapeutic decision-making. Questions range from how comprehensively the cancer genome should be characterized to identify mutated genes that may result in response to a drug, to more social issues regarding physician education and sharing of results. One important question is whether to test only a small number of ‘druggable’ genes for mutations (a panel), or to take an unbiased look across the exome (the DNA sequence of the exons of all known genes in the genome), or to sequence the whole genome. Although the latter is most expensive and time-consuming, whole-genome data can be mined for the multitude of alterations that commonly occur in cancer genomes, including structural variants such as chromosomal translocations, large-scale rearrangements and deletions that often fuse exons to create new fusion proteins. Sequencing the exome or a smaller gene list provides a reduced view of the somatic landscape, has limited capability to capture structural variants, and omits regions such as known promoters or other regulatory regions (for example, the *TERT* gene promoter, a recently discovered mutated driver in melanoma and other tumor types [7,8]). Obviously, the fewer the number of genes studied, the more inexpensive the assay and the more rapid the analysis. In this scenario, however, genes with potential therapeutic indications, such as those in the same biological pathway as a druggable gene that is not itself mutated, will be missed. Hence, a proportion of patients will not obtain a therapeutic match owing to the limited scope of a panel test.

Unfortunately, the complexity of whole-genome analysis, the cost of data generation, and the need for analysis and interpretation to happen in a clinically relevant timeframe have limited the scaling of this most comprehensive approach to high throughput. Our current approach is to sequence the whole genomes of tumor and normal tissue, and to perform exome sequencing of tumor and normal tissue, supplementing the exome capture reagent with additional probe sets that enhance the coverage of frequently mutated genes. This integrated approach improves our ability to detect known variants while not eliminating our ability to discover new variants as well.

A related issue that reflects the difference between PCR-based and massively parallel sequencing (MPS) involves the sequencing of matched normal tissue from the patient. In the existing clinical assay paradigm, only hotspot mutations (such as those discussed earlier in the EGFR tyrosine kinase domain) are sequenced from tumor tissue only. No matching normal tissue is sequenced because these mutations are typically only present in cancer cell genomes, and because the assays are quite focused in their scope, detecting mutations only in restricted, commonly mutated regions. By contrast, the broader scope and ease of obtaining MPS data have changed the focused nature of these assays, resulting in the detection of all variants, somatic and germline, from tumor DNA. Hence, the comparator normal sequence is critically important to define precisely which variants are somatic, but including the normal tissue doubles the cost of sequencing and disrupts the existing clinical assay paradigm. Further complications arise from the broad scope of MPS inquiries because novel mutations in cancer genes are identified (not just the hotspots), often with no clear indication of their pathogenicity (that is, gain or loss of function) or their likely drug response.

Another issue involves a second data type that is proving to be critically informative but is not presently being utilized by most diagnostic testing assays: RNA sequencing of the tumor. RNA sequence data can provide an incredible range of information that identifies genes that are aberrantly overexpressed (often without any indication from DNA sequence data, such as copy number amplification), alleles that are mutated in DNA and potentially druggable, yet are silenced in RNA (again, often without DNA evidence for silencing), and fusion transcripts [9]. In the clinical setting, it is important to detect overexpression of a ‘driver’ RNA owing to the potential for drug targeting (for example, *HER2* in breast cancer), and often the potential for overexpression cannot be detected simply through the examination of DNA (for example, when overexpression is due to altered methylation or histone binding). Furthermore, the support provided by RNA sequence data for analysis of detected DNA variants provides confirmation of potentially druggable mutations. One difficulty in interpreting overexpression lies with the fact that a comprehensive database of RNA expression from RNA sequencing of normal human tissues does not presently exist, although there are efforts to produce such a database (for example, GTEx [10]). When possible, adjacent non-malignant tissue may be sequenced to establish aberrant expression levels in the tumor by comparison. Ultimately, although RNA sequencing represents additional expense and effort, it should enhance our ability to find more answers for more patients [11]. We presently include RNA sequencing of the tumor in our sequencing-based approach to detecting druggable alterations.

Heterogeneity across the cancer cell genomes in each patient’s tumor is just beginning to be understood at the level of DNA [12-14]. The sensitivity to detect heterogeneous cancer genomes is enhanced by the digital nature of NGS and may eventually provide prognostic information (for example, if more heterogeneous tumors are found to be more likely to acquire therapeutic resistance or to metastasize). Although some studies have predicted that heterogeneity may also imply that therapeutic response in patients with multiple metastatic sites will be unsuccessful [12,15], it remains to be seen whether novel, metastasis-specific mutations are common in all tumor types, and indeed whether they are drivers of metastasis. Furthermore, an ultimate aim of translating cancer genomics to clinical care should be to include sequencing-based diagnostic assays at first diagnosis rather than in the advanced disease setting. In this way, current concerns about the complexity of metastatic presentation could well be obviated if the clinical efficacy of precision cancer therapeutics was successful at primary disease presentation. This practice would also enable the detection of recurrent disease or therapeutic resistance with higher sensitivity than provided by available imaging technologies, using blood-based monitoring, as discussed below.

Educational issues remain and must be addressed if physicians are expected to order, understand and act upon diagnostic results obtained from NGS data. These issues are more readily addressed for physicians in training, and most top-tier medical schools are already offering first-year students an introduction to genomic medicine concepts, as well as specialized residency training programs in translational genomics. However, the larger number of practicing physicians who also want to use genomics-based diagnostics but feel alienated because they do not understand the approach and how best to interpret the results of data analysis will need effective continuing medical education (CME)-based training and user-friendly decision support tools to feel sufficiently educated and confident to order and interpret these tests. Furthermore, they will need evidence that specific gene-mutation-targeted therapy combinations will provide clinical benefits, which may be challenging to demonstrate with the current paradigm of one-size-fits-all clinical trials.

A final issue for debate is that no rapid and straightforward mechanism currently exists to communicate the results of what are essentially single-patient studies, often referred to as ‘N of 1’. In essence, early efforts that use NGS diagnostics to aid in therapeutic decision-making will be experimental and will often involve single patients. Analytical and interpretational pipelines will determine which targeted therapies are most likely to benefit each patient, and in the cases where a patient is treated with a targeted therapy and is monitored to evaluate their response, we need a reporting mechanism that can serve to effectively communicate these results to all groups engaged in this enterprise. This type of reporting must include sharing of those cases for which all indications pointed to a specific therapy yet the patient did not achieve a response. Although the typical reporting of case studies occurs through a peer review process, the need for data sharing in this realm is too urgent to wait for the time required to write and publish a manuscript, and the numbers of cases too large to report each one in this manner. Reporting cases from N of 1 studies in an accessible, community-friendly database format will ensure that each patient’s diagnosis and treatment will be as informed as possible. A BioRΧiv-like pre-print server [16] may be worth considering as a vehicle for communication of results. The acquired data from such an exercise would still need to be organized for rapid analysis of new patient data; this is perhaps best funded by the National Institutes of Health (NIH) to ensure free and open access to data while maintaining precautions about data privacy. Those who report cases to the database would have an obligation to follow up on patient outcome data, ensuring maximum benefit. Only as a community willing to share openly will we effectively build the case for the clinical efficacy of genome-guided therapy decisions.

## Practical barriers to translation

There are four main areas that are practical barriers to the successful translation of NGS-based diagnoses for cancer. These relate to tissue preparation methods and their impact on nucleic acids, the limited amount and availability of clinical samples for genomic study, and the clinical trials paradigm - as it affects the approval and availability of targeted therapies. Chief among these is the established practice of preserving tissues in formalin and paraffin. Formalin is problematic because it causes nucleic acid degradation by crosslinking to both DNA and RNA backbones, an effect that is worsened by storage time. There are other preservatives besides formalin, such as HOPE or PAXgene, that are commercially available and are less damaging to nucleic acids while maintaining tissue morphology for microscopic assays. However, supplanting the standard pathology preparatory method of formalin fixation and paraffin embedding is unlikely in the near term because it has been the standard in pathology for over 100 years. Perhaps the best driver of this change would be the widespread use of NGS methods in the diagnostic laboratory. For the time being, methods to determine the extent of degradation of DNA or RNA isolated from formalin/paraffin-preserved samples isolated from a tumor of interest are critical precursors to determine whether these nucleic acids are appropriate for NGS.

Often, in addition to the problems associated with formalin fixation and paraffin embedding, the amount of tissue that is available for nucleic acid isolation is limited. After all, the biopsy will also be needed for conventional diagnostic tests, so it needs to be shared across multiple tests for comprehensive diagnosis. The availability of only low amounts of input nucleic acid is common, and methods tailored to low-input library generation prior to NGS are required. In reality, the combination of formalin/paraffin preservation and low input is quite common, so robust protocols must be designed for the combination of these two limiting factors. Of course, the advantage of using NGS methods is that multiple genes can be studied in a single assay, in contrast to PCR-based assays that evaluate one gene hotspot at a time. With PCR, multiple assays are required to test multiple genes in hopes of identifying a therapeutic match, and if insufficient material is available to run multiple assays, a best guess at which genes are most likely mutated is required.

Radiation therapy and some chemotherapeutic agents damage DNA, and all therapies profoundly influence the genomic heterogeneity of any recurrent or metastatic tumor. As a result, for patients who have failed first- or second-line therapy for their tumor type, a new tumor biopsy must be obtained for genomic studies because the genomic profile of the tumor is likely to have been altered by therapeutic intervention. In a few tumor types, the repeat sampling of metastatic or recurrent disease is straightforward, as it is the standard of care and/or is covered by insurance. Far too often, however, repeat sampling is neither the standard of care nor straightforward (for example, for bone or brain metastases), thereby significantly complicating the ability to assay recent tumor samples to identify new therapeutic targets. New approaches to blood-based monitoring of tumor DNA or cells may help to address these issues, as discussed below.

The fourth practical issue is frequently encountered with NGS-based assays. NGS data often predict that a therapy not approved by the US Food and Drug Administration (FDA) or similar governmental bodies for use in that particular tumor type will be effective. This result reflects the growing data from large-scale genomic discovery projects that show that genes that are frequently mutated in cancers are largely not specific for the tissue type of the tumor [17,18]. Although tissue biology remains important for understanding tumor pathology and drug response, the same somatically altered genes can be initiators of oncogenesis in different tissue types. Hence, a new paradigm for clinical trials of antineoplastic drugs has emerged that hopefully will feed forward into the FDA approval process. One such type of trial is the ‘basket’ trial, in which the gene(s) targeted by a specific drug are tested across patients with multiple disease sites (for instance, lung, colon, breast) and only those patients with a mutational profile suggestive of a therapeutic response are included in the trial [19]. This approach can enable more rapid trial closure or acceleration than trials focusing on a specific disease site, depending upon patient response rates, and could permit FDA consideration of a drug for approval in multiple disease sites [20]. Another trial type is the ‘umbrella’ or ‘master protocol’ trial, in which a collection of targeted therapies and corresponding NGS-based molecular profiling companion diagnostic assays are combined to treat either a single cancer type or multiple cancer types [21]. There are numerous examples of basket and umbrella trials being conducted by pharmaceutical companies, the NIH (such as NCI-MATCH), and by hospital systems (for example, Princess Margaret Hospital’s IMPACT trial [22]).

## Promising new translational approaches

As frequently occurs with emerging technology, innovative approaches that address several of the aforementioned issues are rapidly coming onto the scene. For example, in the case of acquired resistance to therapy, earlier detection is critical to permit the oncologist to devise a new therapeutic regimen before metastatic disease is too advanced. However, metastatic disease is often detected too late by imaging technologies that require a tumor mass to achieve a minimal size before it can be detected. New efforts are pursuing the early detection of circulating tumor cells (CTCs) or circulating DNA or RNA [23], often using NGS to characterize the initial disease presentation, so that mutated genes are identified for that patient’s disease and can then be detected by less expensive, more rapid assays using blood samples, taken at strategic time points after surgery to remove the tumor [24,25]. As the genomic alterations that emerge in resistance to targeted therapies are better understood [26-28], these also may be monitored by specific blood-based assays. Another example of an application for CTC- or circulating nucleic-acid-based monitoring is in prostate cancer. This approach is currently being used to monitor the progression to androgen-resistant disease, which typically forms metastases in bone [29,30]. However, the approach could also be used as a means of informing active surveillance for men with a prostate cancer diagnosis but low risk of developing aggressive disease.

The basket and umbrella clinical trial designs will identify more cases of ‘extreme responses’ to targeted therapies, defined as those patients whose tumor burden is alleviated rapidly (complete remission) and, in some cases, with long duration [31]. Comprehensive genomic studies of these patients may add genes to the repertoire of diagnostic assays that predict the likelihood of extreme response, and thus better inform therapeutic decision-making. A related consequence of genomic testing for therapy selection is the likely transition of companion diagnostics to sequence-based tests, rather than slower, less sensitive and less specific assays such as fluorescent *in situ* hybridization (FISH) or cytogenetics. As a result, more patients will be correctly identified as candidates for the drugs that are most likely to help relieve their disease burden.

One interesting application of NGS may amplify the resurgence of immunotherapies, which is currently being driven by the antineoplastic properties of the new checkpoint blockade agents that invoke the immune system’s interaction with cancer cells [32-36]. In a new paradigm, NGS identifies tumor-specific mutations and their RNA expression levels, and these data are used to predict immunoepitopes for vaccine development, ignoring the druggable targets and instead prioritizing those tumor-unique peptides that are most immune-stimulatory, for use in vaccine development [37,38]. An algorithmic method for prediction of class I human leukocyte antigen (HLA) binding affinity considers the highly expressed somatic missense (altering the amino acid sequence) mutations discovered by NGS analysis, and provides scores for the binding affinity of mutant neoantigens compared with their corresponding wild-type protein sequences [39]. Various *in vitro* tests using patient-derived T cells can further refine the list of candidates for use in personalized vaccine development, to provide a final list of the most immune-stimulatory peptide sequences that are unique to a patient’s tumor. A variety of approaches can be considered for producing a patient-specific vaccine, such as a multivalent DNA vaccine that includes the prioritized peptide-encoding sequences in a vaccine vector, or a dendritic-cell vaccine that uses patient-specific peptides to stimulate the patient’s own dendritic cells to induce T-cell memory [37]. Furthermore, the astounding sequencing capacity of NGS instruments now makes monitoring changes in a patient’s T- and B-cell repertoires quite straightforward within a single experiment [40], and could provide a critically important component of patient monitoring during immunotherapy or other therapeutic interventions.

## Conclusions and future directions

Multiple technological innovations fueled by the application of NGS technology to cancer genomics are now making the clinical use of NGS platforms and assays increasingly common. Applications for therapeutic decision-making, including for new clinical trials of targeted therapies, are increasing the demand for NGS assays and improving cancer care and patient monitoring. Over coming years, this virtuous circle will provide important metrics that establish the sensitivity and specificity of NGS-based diagnostic assays in comparison to current clinical standards (for example, FISH or immunohistochemistry). These efforts will also consolidate the use of NGS-based diagnostics in cancer medicine by virtue of clinical efficacy, as the precision afforded by these tests results in patient relief from disease burden by prescribing the right drug for each patient’s disease. The associated disruption caused by these diagnostic changes will affect current pathology-based diagnoses, challenge standards of care for most cancer types, and invoke the many other issues discussed here. Ultimately, this disruption and the resolution of these issues will benefit patients by ensuring more precise diagnostic and therapeutic results in their fight against cancer.

## Abbreviations

CTC: circulating tumor cell; FDA: Food and Drug Administration; FISH: fluorescent *in situ* hybridization; MPS: massively parallel sequencing; NGS: next-generation sequencing; TKI: tyrosine kinase inhibitor.

## Competing interests

The author declares that they have no competing interests.

## References

[B1] International Human Genome Sequencing Consortium Finishing the euchromatic sequence of the human genome Nature 2004 431 931 945 10.1038/nature03001 15496913

[B2] Pao W Miller V Zakowski M Doherty J Politi K Sarkaria I Singh B Heelan R Rusch V Fulton L Mardis E Kupfer D Wilson R Kris M Varmus H EGF receptor gene mutations are common in lung cancers from “never smokers” and are associated with sensitivity of tumors to gefitinib and erlotinib Proc Natl Acad Sci USA 2004 101 13306 13311 10.1073/pnas.0405220101 15329413 PMC516528

[B3] Lynch TJ Bell DW Sordella R Gurubhagavatula S Okimoto RA Brannigan BW Harris PL Haserlat SM Supko JG Haluska FG Louis DN Christiani DC Settleman J Haber DA Activating mutations in the epidermal growth factor receptor underlying responsiveness of non-small-cell lung cancer to gefitinib N Engl J Med 2004 350 2129 2139 10.1056/NEJMoa040938 15118073

[B4] Paez JG Jänne PA Lee JC Tracy S Greulich H Gabriel S Herman P Kaye FJ Lindeman N Boggon TJ Naoki K Sasaki H Fujii Y Eck MJ Sellers WR Johnson BE Meyerson M EGFR mutations in lung cancer: correlation with clinical response to gefitinib therapy Science 2004 304 1497 1500 10.1126/science.1099314 15118125

[B5] Pao W Hutchinson KE Chipping away at the lung cancer genome Nat Med 2012 18 349 351 10.1038/nm.2697 22395697

[B6] Koboldt DC Steinberg KM Larson DE Wilson RK Mardis ER The next-generation sequencing revolution and its impact on genomics Cell 2013 155 27 38 10.1016/j.cell.2013.09.006 24074859 PMC3969849

[B7] Horn S Figl A Rachakonda PS Fischer C Sucker A Gast A Kadel S Moll I Nagore E Hemminki K Schadendorf D Kumar R TERT promoter mutations in familial and sporadic melanoma Science 2013 339 959 961 10.1126/science.1230062 23348503

[B8] Huang FW Hodis E Xu MJ Kryukov GV Chin L Garraway LA Highly recurrent TERT promoter mutations in human melanoma Science 2013 339 957 959 10.1126/science.1229259 23348506 PMC4423787

[B9] Mutz KO Heilkenbrinker A Lönne M Walter JG Stahl F Transcriptome analysis using next-generation sequencing Curr Opin Biotechnol 2013 24 22 30 10.1016/j.copbio.2012.09.004 23020966

[B10] Genotype-Tissue Expression (GTEx) project. [http://commonfund.nih.gov/GTEx/index]

[B11] Govindan R Ding L Griffith M Subramanian J Dees ND Kanchi KL Maher CA Fulton R Fulton L Wallis J Chen K Walker J McDonald S Bose R Ornitz D Xiong D You M Dooling DJ Watson M Mardis ER Wilson RK Genomic landscape of non-small cell lung cancer in smokers and never-smokers Cell 2012 150 1121 1134 10.1016/j.cell.2012.08.024 22980976 PMC3656590

[B12] Gerlinger M Rowan AJ Horswell S Larkin J Endesfelder D Gronroos E Martinez P Matthews N Stewart A Tarpey P Varela I Phillimore B Begum S McDonald NQ Butler A Jones D Raine K Latimer C Santos CR Nohadani M Eklund AC Spencer-Dene B Clark G Pickering L Stamp G Gore M Szallasi Z Downward J Futreal PA Swanton C Intratumor heterogeneity and branched evolution revealed by multiregion sequencing N Engl J Med 2012 366 883 892 10.1056/NEJMoa1113205 22397650 PMC4878653

[B13] Ding L Ley TJ Larson DE Miller CA Koboldt DC Welch JS Ritchey JK Young MA Lamprecht T McLellan MD McMichael JF Wallis JW Lu C Shen D Harris CC Dooling DJ Fulton RS Fulton LL Chen K Schmidt H Kalicki-Veizer J Magrini VJ Cook L McGrath SD Vickery TL Wendl MC Heath S Watson MA Link DC Tomasson MH Clonal evolution in relapsed acute myeloid leukaemia revealed by whole-genome sequencing Nature 2012 481 506 510 10.1038/nature10738 22237025 PMC3267864

[B14] Nik-Zainal S Van Loo P Wedge DC Alexandrov LB Greenman CD Lau KW Raine K Jones D Marshall J Ramakrishna M Shlien A Cooke SL Hinton J Menzies A Stebbings LA Leroy C Jia M Rance R Mudie LJ Gamble SJ Stephens PJ McLaren S Tarpey PS Papaemmanuil E Davies HR Varela I McBride DJ Bignell GR Leung K Butler AP The life history of 21 breast cancers Cell 2012 149 994 1007 10.1016/j.cell.2012.04.023 22608083 PMC3428864

[B15] Murugaesu N Chew SK Swanton C Adapting clinical paradigms to the challenges of cancer clonal evolution Am J Pathol 2013 182 1962 1971 10.1016/j.ajpath.2013.02.026 23708210

[B16] Biorxiv [http://biorxiv.org/about-biorxiv]

[B17] Kandoth C McLellan MD Vandin F Ye K Niu B Lu C Xie M Zhang Q McMichael JF Wyczalkowski MA Leiserson MD Miller CA Welch JS Walter MJ Wendl MC Ley TJ Wilson RK Raphael BJ Ding L Mutational landscape and significance across 12 major cancer types Nature 2013 502 333 339 10.1038/nature12634 24132290 PMC3927368

[B18] Weinstein JN Collisson EA Mills GB Shaw KR Ozenberger BA Ellrott K Shmulevich I Sander C Stuart JM Cancer Genome Atlas Research Network The Cancer Genome Atlas Pan-Cancer analysis project Nat Genet 2013 45 1113 1120 10.1038/ng.2764 24071849 PMC3919969

[B19] Sleijfer S Bogaerts J Siu LL Designing transformative clinical trials in the cancer genome era J Clin Oncol 2013 31 1834 1841 10.1200/JCO.2012.45.3639 23589555

[B20] Willyard C ‘Basket studies’ will hold intricate data for cancer drug approvals Nat Med 2013 19 655 10.1038/nm0613-655 23744135

[B21] Ledford H ‘Master protocol’ aims to revamp cancer trials Nature 2013 498 146 147 10.1038/498146a 23765467

[B22] Princess Margaret Hospital’s IMPACT trial. [http://thepmcf.ca/Pages/ItsPersonal/ItsPersonal.Story.aspx?s=195]

[B23] Crowley E Di Nicolantonio F Loupakis F Bardelli A Liquid biopsy: monitoring cancer-genetics in the blood Nat Rev Clin Oncol 2013 10 472 484 10.1038/nrclinonc.2013.110 23836314

[B24] Leary RJ Sausen M Kinde I Papadopoulos N Carpten JD Craig D O’Shaughnessy J Kinzler KW Parmigiani G Vogelstein B Diaz LA Jr Velculescu VE Detection of chromosomal alterations in the circulation of cancer patients with whole-genome sequencing Sci Transl Med 2012 4 162ra154 23197571 10.1126/scitranslmed.3004742 PMC3641759

[B25] Dawson SJ Rosenfeld N Caldas C Circulating tumor DNA to monitor metastatic breast cancer N Engl J Med 2013 369 93 94 23822788 10.1056/NEJMc1306040

[B26] Sequist LV Waltman BA Dias-Santagata D Digumarthy S Turke AB Fidias P Bergethon K Shaw AT Gettinger S Cosper AK Akhavanfard S Heist RS Temel J Christensen JG Wain JC Lynch TJ Vernovsky K Mark EJ Lanuti M Iafrate AJ Mino-Kenudson M Engelman JA Genotypic and histological evolution of lung cancers acquiring resistance to EGFR inhibitors Sci Transl Med 2011 3 75ra26 21430269 10.1126/scitranslmed.3002003 PMC3132801

[B27] Lo RS Receptor tyrosine kinases in cancer escape from BRAF inhibitors Cell Res 2012 22 945 947 10.1038/cr.2012.78 22565288 PMC3367528

[B28] Lo RS Shi H Detecting mechanisms of acquired BRAF inhibitor resistance in melanoma Methods Mol Biol 2014 1102 163 174 10.1007/978-1-62703-727-3_10 24258979

[B29] Perkins G Yap TA Pope L Cassidy AM Dukes JP Riisnaes R Massard C Cassier PA Miranda S Clark J Denholm KA Thway K Gonzalez De Castro D Attard G Molife LR Kaye SB Banerji U de Bono JS Multi-purpose utility of circulating plasma DNA testing in patients with advanced cancers PLoS One 2012 7 e47020 10.1371/journal.pone.0047020 23144797 PMC3492590

[B30] Ligthart ST Coumans FA Bidard FC Simkens LH Punt CJ de Groot MR Attard G de Bono JS Pierga JY Terstappen LW Circulating tumor cells count and morphological features in breast, colorectal and prostate cancer PLoS One 2013 8 e67148 10.1371/journal.pone.0067148 23826219 PMC3695007

[B31] Iyer G Hanrahan AJ Milowsky MI Al-Ahmadie H Scott SN Janakiraman M Pirun M Sander C Socci ND Ostrovnaya I Viale A Heguy A Peng L Chan TA Bochner B Bajorin DF Berger MF Taylor BS Solit DB Genome sequencing identifies a basis for everolimus sensitivity Science 2012 338 221 10.1126/science.1226344 22923433 PMC3633467

[B32] Topalian SL Sznol M McDermott DF Kluger HM Carvajal RD Sharfman WH Brahmer JR Lawrence DP Atkins MB Powderly JD Leming PD Lipson EJ Puzanov I Smith DC Taube JM Wigginton JM Kollia GD Gupta A Pardoll DM Sosman JA 2014 Survival, durable tumor remission, and long-term safety in patients with advanced melanoma receiving nivolumab. J Clin Oncol: Hodi FS doi:10.1200/JCO.2013.53.0105 10.1200/JCO.2013.53.0105 PMC4811023 24590637

[B33] Brahmer JR Tykodi SS Chow LQ Hwu WJ Topalian SL Hwu P Drake CG Camacho LH Kauh J Odunsi K Pitot HC Hamid O Bhatia S Martins R Eaton K Chen S Salay TM Alaparthy S Grosso JF Korman AJ Parker SM Agrawal S Goldberg SM Pardoll DM Gupta A Wigginton JM Safety and activity of anti-PD-L1 antibody in patients with advanced cancer N Engl J Med 2012 366 2455 2465 10.1056/NEJMoa1200694 22658128 PMC3563263

[B34] Topalian SL Hodi FS Brahmer JR Gettinger SN Smith DC McDermott DF Powderly JD Carvajal RD Sosman JA Atkins MB Leming PD Spigel DR Antonia SJ Horn L Drake CG Pardoll DM Chen L Sharfman WH Anders RA Taube JM McMiller TL Xu H Korman AJ Jure-Kunkel M Agrawal S McDonald D Kollia GD Gupta A Wigginton JM Sznol M Safety, activity, and immune correlates of anti-PD-1 antibody in cancer N Engl J Med 2012 366 2443 2454 10.1056/NEJMoa1200690 22658127 PMC3544539

[B35] Mitsui J Nishikawa H Muraoka D Wang L Noguchi T Sato E Kondo S Allison JP Sakaguchi S Old LJ Kato T Shiku H Two distinct mechanisms of augmented antitumor activity by modulation of immunostimulatory/inhibitory signals Clin Cancer Res 2010 16 2781 2791 10.1158/1078-0432.CCR-09-3243 20460483

[B36] Pedicord VA Montalvo W Leiner IM Allison JP Single dose of anti-CTLA-4 enhances CD8^+^ T-cell memory formation, function, and maintenance Proc Natl Acad Sci USA 2011 108 266 271 10.1073/pnas.1016791108 21173239 PMC3017182

[B37] Carreno BM Becker-Hapak M Huang A Chan M Alyasiry A Lie WR Aft RL Cornelius LA Trinkaus KM Linette GP IL-12p70-producing patient DC vaccine elicits Tc1-polarized immunity J Clin Invest 2013 123 3383 3394 10.1172/JCI68395 23867552 PMC3726168

[B38] van Rooij N van Buuren MM Philips D Velds A Toebes M Heemskerk B van Dijk LJ Behjati S Hilkmann H El Atmioui D Nieuwland M Stratton MR Kerkhoven RM Kesmir C Haanen JB Kvistborg P Schumacher TN Tumor exome analysis reveals neoantigen-specific T-cell reactivity in an ipilimumab-responsive melanoma J Clin Oncol 2013 31 e439 e442 10.1200/JCO.2012.47.7521 24043743 PMC3836220

[B39] Lundegaard C Lamberth K Harndahl M Buus S Lund O Nielsen M NetMHC-3.0: accurate web accessible predictions of human, mouse and monkey MHC class I affinities for peptides of length 8–11 Nucleic Acids Res 2008 36 W509 W512 10.1093/nar/gkn202 18463140 PMC2447772

[B40] Mori A Deola S Xumerle L Mijatovic V Malerba G Monsurrò V Next generation sequencing: new tools in immunology and hematology Blood Res 2013 48 242 249 10.5045/br.2013.48.4.242 24466547 PMC3894381

